# Intravital imaging of peritubular microcirculation impairment in cisplatin-induced acute kidney injury

**DOI:** 10.1172/jci.insight.178689

**Published:** 2025-04-08

**Authors:** Inwon Park, Seonghye Kim, Young Woo Um, Hee Eun Kim, Jae Hyuk Lee, Sejoong Kim, Pilhan Kim, You Hwan Jo

**Affiliations:** 1 Department of Emergency Medicine, Seoul National University Bundang Hospital (SNUBH), Seongnam, Republic of Korea.; 2 Department of Emergency Medicine, and; 3 Disaster Medicine Research Center, Seoul National University Medical Research Center, Seoul National University College of Medicine, Seoul, Republic of Korea.; 4 Division of Nephrology, Department of Internal Medicine, Seoul National University Bundang Hospital, Seongnam, Republic of Korea.; 5 Department of Internal Medicine, Seoul National University College of Medicine, Seoul, Republic of Korea.; 6 Graduate School of Medical Science and Engineering, and; 7 KI for Health Science and Technology (KIHST), Korea Advanced Institute of Science and Technology (KAIST), Daejeon, Republic of Korea.

**Keywords:** Nephrology, Vascular biology, Chronic kidney disease, Diagnostic imaging, Microcirculation

## Abstract

Despite the accumulation of cisplatin in proximal tubules, direct visualization of the surrounding peritubular microcirculation, including its change in cisplatin-induced acute kidney injury (AKI), is lacking. Here, using fluorescence and cellular angiography through video-rate high-resolution intravital microscopy, progressive disturbance of peritubular microcirculation in cisplatin-induced AKI in mice was demonstrated. Fluorescence angiography revealed increasing perfusion defects, with a stepwise rise in time to peak (TTP), originating from capillaries surrounding S1 segments. Cellular angiography demonstrated a progressive decrease in the velocity and track length of individual erythrocytes during AKI progression, accompanied by a sequential decrease in the functional capillary ratio (FCR). Changes in the perfusion area, TTP, and FCR preceded significant changes in blood urea nitrogen and cystatin C, suggesting the potential for early diagnosis. Although neutrophil infiltration near proximal tubules increased throughout the progression, it did not cause obstruction of the peritubular microcirculation. Depletion of neutrophils increased mortality due to systemic side effects, whereas functional inactivation of neutrophils using an anti-CD11b antibody improved peritubular microcirculation in cisplatin-induced AKI. This approach enables direct visualization and quantification of peritubular microcirculation and immune cell dynamics, providing insights into renal pathophysiology and potential therapeutic strategies.

## Abstract 2

Using intravital microscopy combined with fluorescence and cellular angiography, we directly visualized and quantified the peritubular microcirculation in cisplatin-induced AKI.

## Graphical abstract

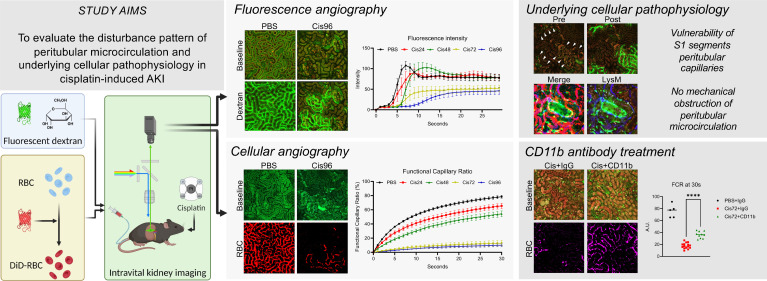

## Introduction

Cisplatin is a highly potent and effective chemotherapy that has been extensively used for treating various cancers (1, 2). Notwithstanding the potent antineoplastic effectiveness of cisplatin, its clinical utilization is restricted due to severe side effects and dose-dependent nephrotoxicity (1, 3). The key molecular mechanisms in cisplatin-induced acute kidney injury (AKI) involve the cellular uptake and accumulation of cisplatin in proximal tubule epithelial cells (4–6). These cells are surrounded by the peritubular capillary system, which provides the necessary oxygen and nutrients and maintains fluid and electrolyte balance (7). Despite its vital importance in preserving renal function as a major capillary network in the kidney, a comprehensive understanding of the microcirculation in cisplatin-induced AKI has not been fully elucidated (8, 9). Moreover, in addition to its direct cytotoxic effects, cisplatin triggers an inflammatory cascade that stimulates the production of inflammatory mediators by both renal parenchymal cells and resident or infiltrating leukocytes, thereby contributing to AKI (6, 10, 11). Given these findings, direct visualization and quantification of the peritubular microcirculation at the cellular level, including leukocytes, is warranted to understand the mechanisms of cisplatin-induced AKI (1, 12).

Intravital microscopy offers a distinctive opportunity to observe the dynamic behavior of cells in their native environment in live subjects (13). While advances in intravital microscopy have provided comprehensive insight into translational kidney research, investigations including the quantification of microcirculation at the cellular level are somewhat limited (14). Using an intravital microscope with video-rate scanning speed and high resolution down to the cellular level may achieve direct visualization of microcirculation, including quantifiable parameters comparable to each other (15). Given that cisplatin accumulation is known to induce injury in proximal tubular epithelial cells in cisplatin-induced AKI, it is an optimal approach to investigate the peritubular microcirculation with an additional focus on leukocytes.

Therefore, to evaluate the peritubular microcirculation in cisplatin-induced AKI, we adopted fluorescence and cellular angiography using a video-rate laser scanning confocal microscope. Fluorescence angiography, inspired by the strategy of using contrast agents in computed tomography angiography in clinical settings, enabled spatiotemporal analyses of hemodynamic parameters and perfusion proportion in peritubular capillaries during cisplatin-induced AKI. Cellular angiography via the adoptive transfer of fluorescently tagged erythrocytes allowed quantification of the functional proportion of peritubular capillaries and longitudinal measurement of microcirculation irrespective of the time of fluorescence injection. Here, by using an in vivo kidney imaging platform, we directly visualized how microcirculation is disrupted in the peritubular capillary system in cisplatin-induced AKI and proposed quantitative parameters representing the degree of microcirculation.

## Results

### Fluorescence angiography in cisplatin-induced AKI.

**Figure 1:**
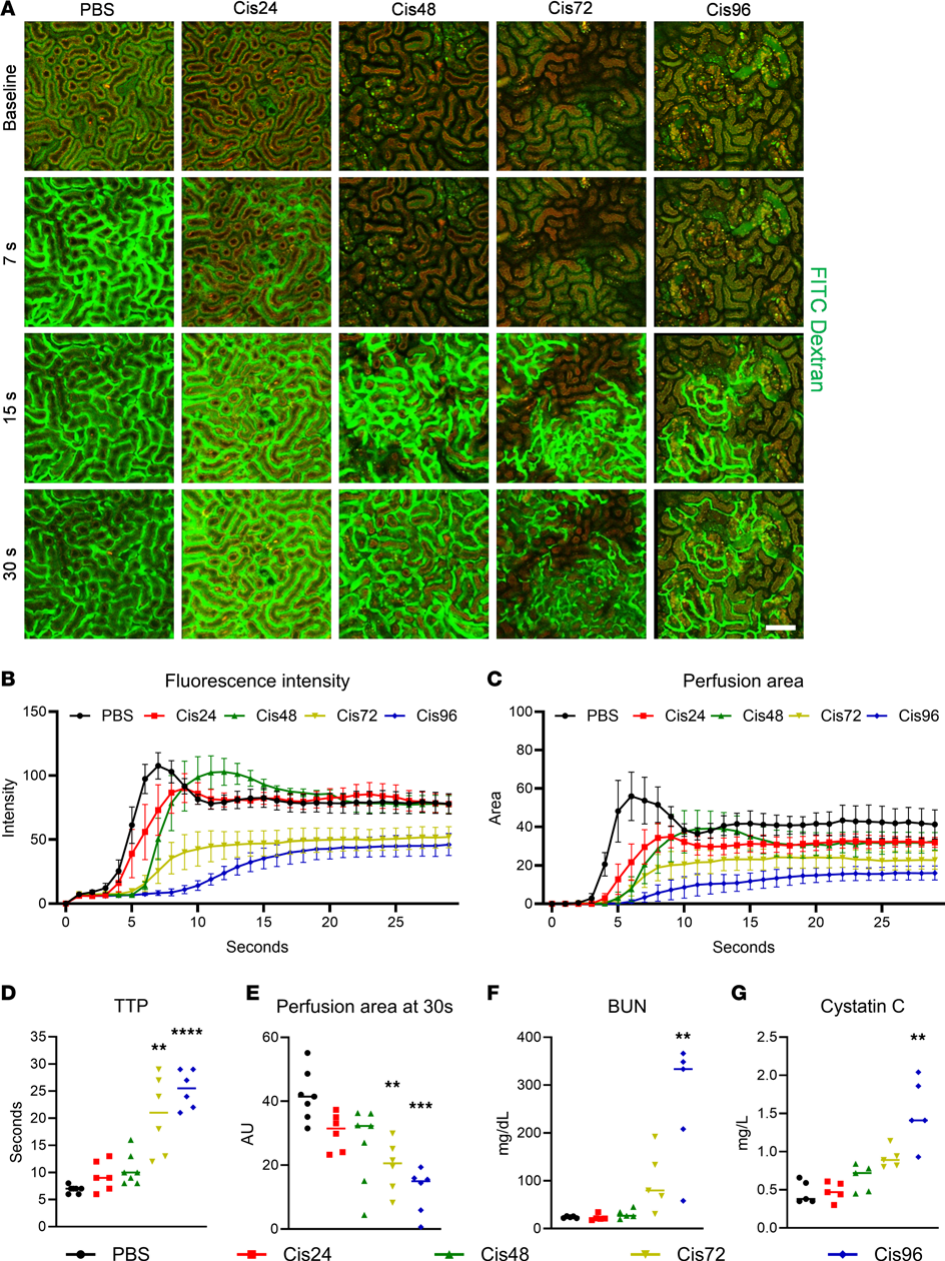
Fluorescence angiography using intravital microscopy in the cisplatin-induced AKI model. (**A**) Representative fluorescence angiography sequential imaging in the PBS, 24-hour, 48-hour, 72-hour, and 96-hour post-cisplatin groups. See also Supplemental Video 1. Scale bar: 100 μm. (**B** and **C**) Time-intensity curve of fluorescence intensity and perfusion area in each group (*n* = 6–7). Data are expressed as the mean ± SD. (**D** and **E**) Comparisons of time-to-peak (TTP) and perfusion area at 30 seconds between groups. (**F** and **G**) Comparisons of biomarkers (BUN and cystatin C) in the cisplatin-induced AKI model. The middle line represents the median value, and statistical significance was assessed using the Kruskal-Wallis test, followed by Dunn’s multiple-comparison test against the PBS group (**D**–**G**). **P < 0.01, ***P < 0.001, ****P < 0.0001.

Fluorescence angiography with FITC-dextran (2,000 kDa) was performed to investigate the peritubular microcirculation in a cisplatin-induced AKI model (Figure 1A and Supplemental Video 1; supplemental material available online with this article; https://doi.org/10.1172/jci.insight.178689DS1). To measure the flow dynamics in the peritubular microcirculation, simultaneous recording at the beginning time of intravenous fluorescence injection was conducted. Compared with the control group (PBS), the increase in fluorescence intensity was sequentially delayed, and perfusing areas gradually decreased in the cisplatin-induced AKI model (Figure 1, B and C). The time to peak (TTP) was significantly increased (Figure 1D), and the perfusion area at 30 seconds decreased in a progressive manner (Figure 1E). Interestingly, 48 hours after cisplatin induction, tubules exhibited 2 distinct phenotypes, one characterized by a high autofluorescence signal. Based on previously established methods (16–18), a time-series test with low molecular weight tetramethylrhodamine-conjugated dextran (TMR-dextran, 4 kDa) was conducted to identify tubular segments exhibiting prominent autofluorescence signal (Supplemental Figure 1 and Supplemental Video 2). The results revealed that the S2 segments of the proximal tubule exhibited the high autofluorescence signal. The peritubular microcirculation near these S2 segments was preserved, while the peritubular microcirculation near the S1 segments was not maintained (Supplemental Figure 2A). Immunostaining with an anti-OAT1 antibody, a selective marker for S2 segments (19, 20), further confirmed that peritubular microcirculation near S1 tubules was compromised (Supplemental Figure 2B and Supplemental Video 3). To determine whether impaired capillary flow resulted from capillary compression due to tubular cell swelling, we analyzed the tubular area. However, instead of an increase, we observed a progressive decrease in tubular area following cisplatin administration, suggesting that factors other than swelling may contribute to the observed flow impairment (Supplemental Figure 3). While blood urea nitrogen (BUN) (Figure 1F) and cystatin C (Figure 1G) showed significant differences from 96 hours after cisplatin, perfusion and TTP exhibited earlier changes in the peritubular microcirculation, beginning at 72 hours.

### Cellular angiography in cisplatin-induced AKI.

**Figure 2:**
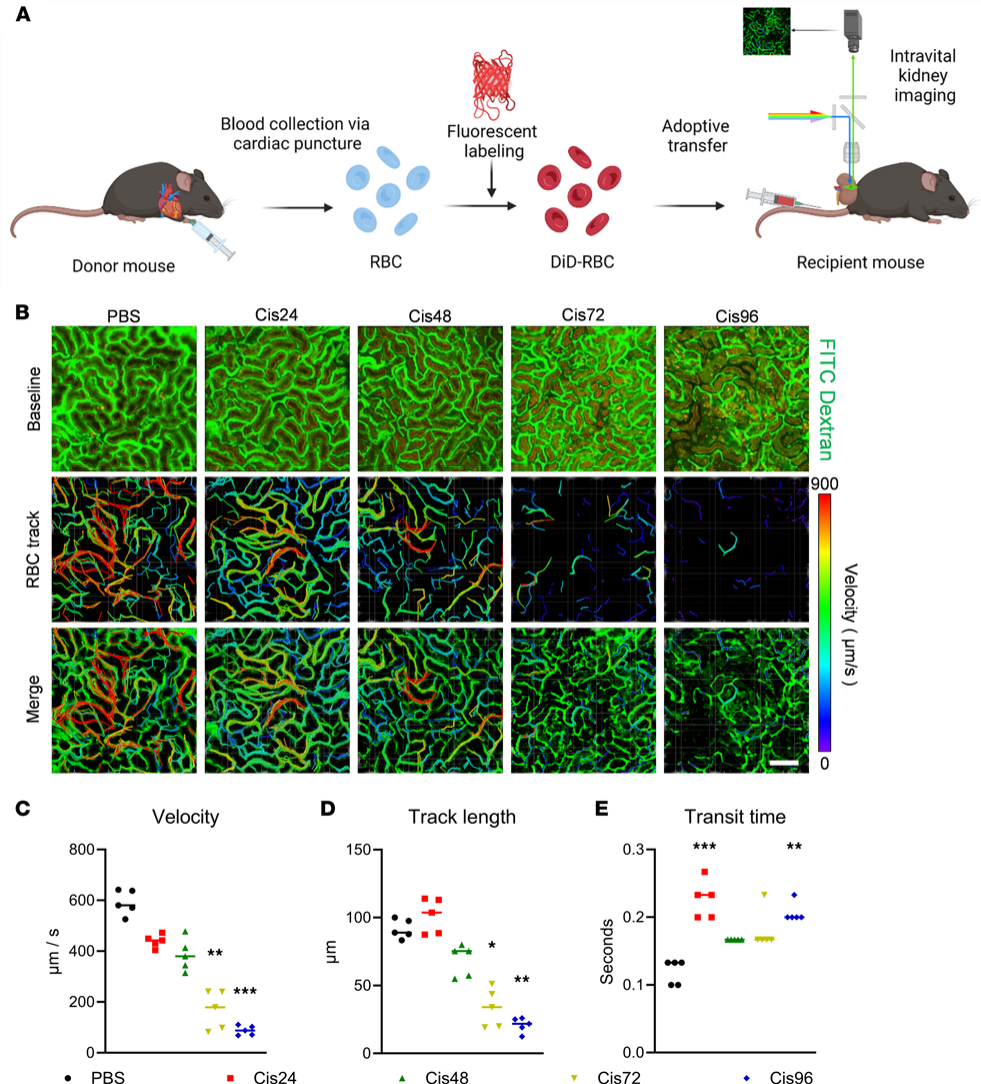
Cellular angiography using adoptive transfer of erythrocytes in intravital microscopy in the cisplatin-induced AKI model. (**A**) Schematics of the adoptive transfer of DiD-labeled erythrocytes into recipient mice, followed by intravital kidney imaging. Created with BioRender.com. (**B**) Representative erythrocyte trajectories in the PBS, 24-hour, 48-hour, 72-hour, and 96-hour post-cisplatin groups. See also Supplemental Video 4. Scale bar: 100 μm. (**C**–**E**) Comparisons of velocity, track length, and transit time between groups. The middle line represents the median value, and statistical significance was assessed using the Kruskal-Wallis test, followed by Dunn’s multiple-comparison test against the PBS group (**C**–**E**). *P < 0.05, **P < 0.01, ***P < 0.001.

The use of fluorescence angiography is limited by the fact that it can be utilized only during the injection period, and further imaging is unfeasible until fluorescent agents are completely washed out from the kidney, similar to the features of computed tomography angiography. To address this technical challenge, we next utilized cellular angiography by using adoptive transfer of erythrocytes (5.0 × 10^7^ cells) labeled with a fluorescent probe (DiD) into recipient mice, as previously described (15) (Figure 2A). Rapidly flowing erythrocytes were clearly visible in real time inside the peritubular capillaries previously labeled with FITC-dextran (Supplemental Video 4). Fluorescently labeled erythrocytes were utilized to acquire spatiotemporal information regarding the trajectories and velocities of individual erythrocytes, enabling in vivo simultaneous tracking of multiple erythrocytes (Figure 2B). Compared with the control group (PBS), both the velocity (Figure 2C) and track length (Figure 2D) progressively decreased, while capillary transit time (Figure 2E) of erythrocytes increased, with a transient elevation observed at 24 hours. Collectively, these observations suggest that the diminished flow of microcirculation led to an extended indwelling time of circulating erythrocytes within the peritubular capillary. To delineate whether this microcirculatory disturbance was derived from consequences of a decline in systemic perfusion, additional imaging of erythrocyte velocity was performed in proximal vessels of the ear (Supplemental Figure 4). Perfusion in the ear vessels, serving as an indicator of systemic perfusion, showed no decrease after cisplatin administration.

### The FCR in cisplatin-induced AKI.

**Figure 3:**
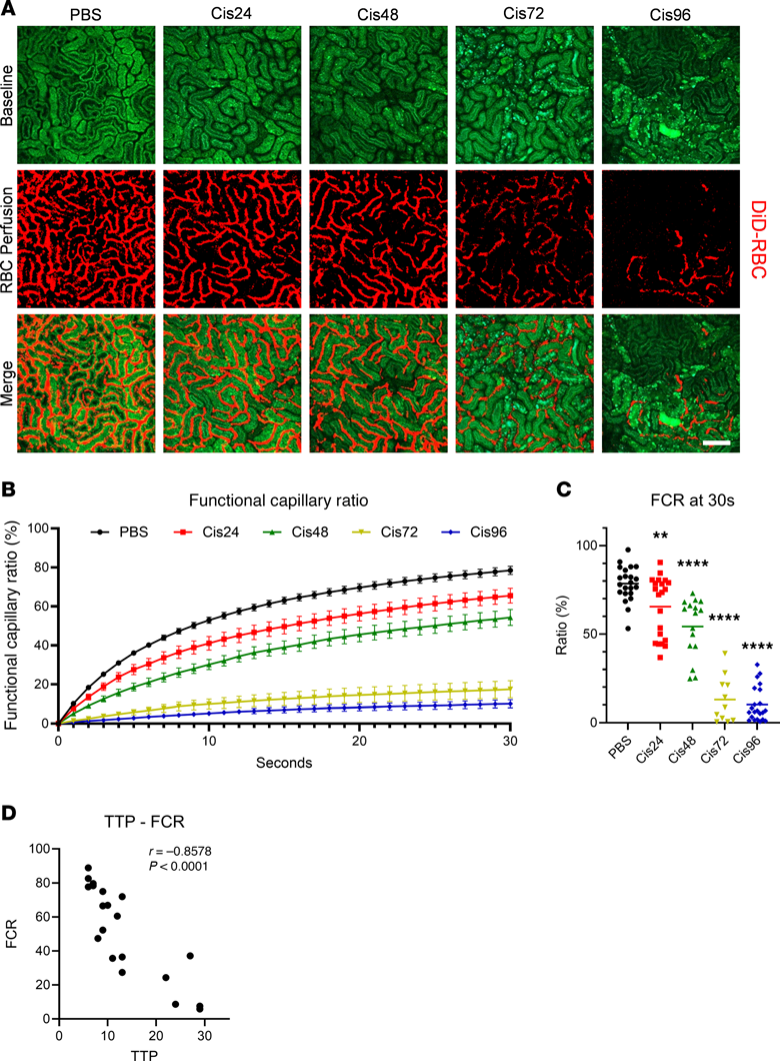
Functional capillary ratio (FCR) using cellular angiography in the cisplatin-induced AKI model. (**A**) Representative functional capillary imaging in the PBS, 24-hour, 48-hour, 72-hour, and 96-hour post-cisplatin groups. See also Supplemental Video 5. Scale bar: 100 μm. (**B**) Time-intensity curves of FCR in each group (*n* = 22 for PBS, *n* = 21 for Cis24, *n* = 16 for Cis48, *n* = 11 for Cis72, *n* = 22 for Cis96). Data are expressed as the mean ± SD. (**C**) Comparison of FCR at 30 seconds in each group. The middle line represents the median value, and statistical significance was assessed using the Kruskal-Wallis test, followed by Dunn’s multiple-comparison test against the PBS group. **P < 0.01, ****P < 0.0001. (**D**) Correlation between time-to-peak (TTP) and FCR, with Pearson’s correlation coefficient (*r*) and significance level indicated.

After individual dynamic analysis, further investigation was performed on erythrocyte dynamics to determine the distribution of functional capillaries in the peritubular microcirculation (Figure 3A and Supplemental Video 5). The functional capillary ratio (FCR) was evaluated based on a previous study (15) and was found to progressively decrease in cisplatin-induced AKI (Figure 3, B and C). Notably, FCR exhibited a significant decrease beginning at 24 hours, while the previously evaluated AKI biomarkers increased significantly from 96 hours after inducing the AKI model with cisplatin. This FCR imaging also confirmed the disproportionate concentration of microcirculation near the S1 segments in proximal tubules with a low autofluorescence signal, coinciding with previous observations in fluorescence angiography (Cis 96 in Figure 3A). In addition, TTP in the fluorescence angiography exhibited strong correlations with FCR (Figure 3D).

### Role of neutrophils in cisplatin-induced AKI.

**Figure 4:**
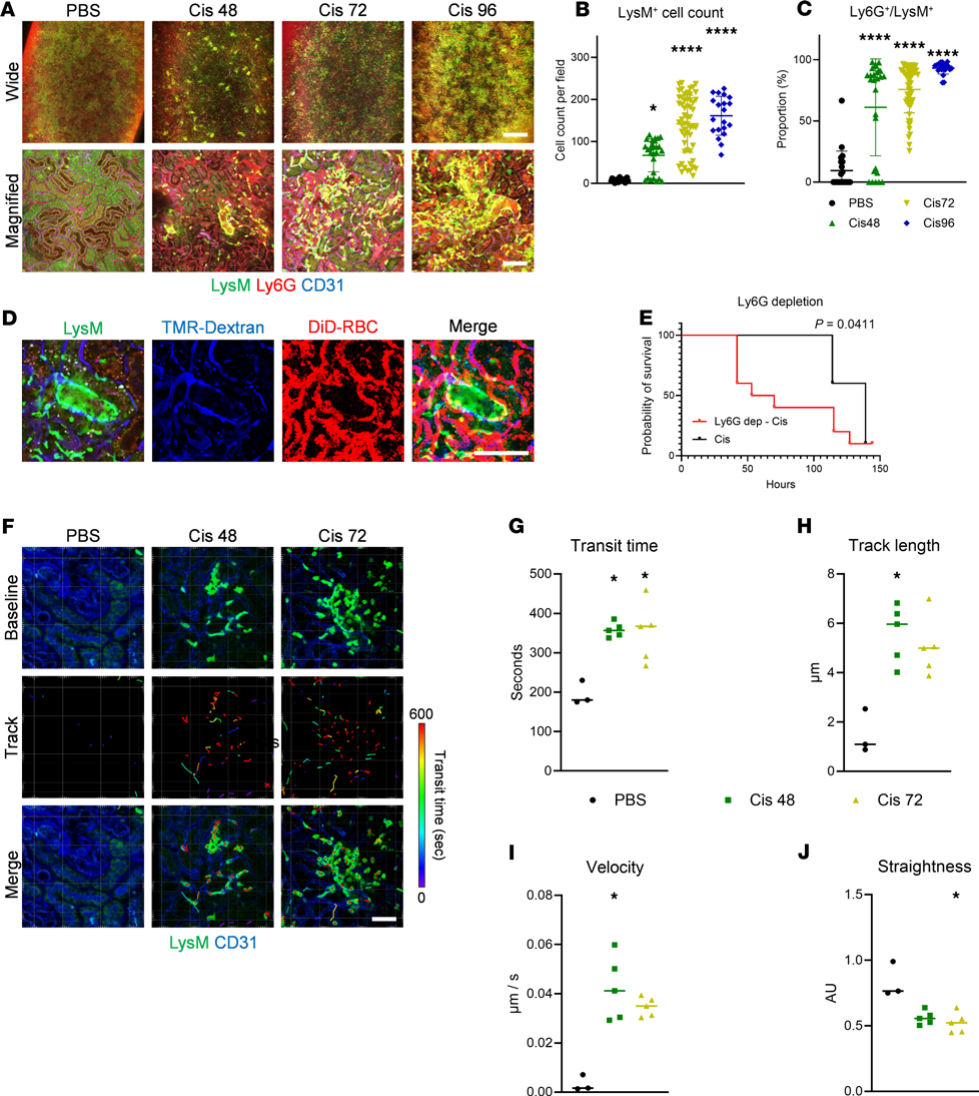
Role of neutrophils in the cisplatin-induced AKI model. (**A**) Representative imaging of *LysM*^GFP/+^ mice in the PBS, 48-hour, 72-hour, and 96-hour post-cisplatin groups. Scale bars: 1 mm (wide) and 100 μm (magnified). (**B** and **C**) Comparisons of LysM^+^ cell count and proportion of Ly6G^+^ cells within the LysM^+^ population. The middle line represents the median value, and statistical significance was assessed using the Kruskal-Wallis test, followed by Dunn’s multiple-comparison test against the PBS group. *P < 0.05, ****P < 0.0001. (**D**) Representative angiography images in *LysM*^gfp/+^ mice, showing that neutrophil infiltration in tubules does not block nearby microcirculation. Scale bar: 100 μm. See also Supplemental Video 6. (**E**) Survival curve of the Ly6G^+^ cell depletion model in cisplatin-induced AKI (*n* = 10 for each group). *P* value indicates the result of the log-rank test. (**F**) Representative images of neutrophil dynamics in the PBS, 48-hour, and 72-hour post-cisplatin groups. Scale bar: 100 μm. See also Supplemental Video 7. (**G**–**J**) Comparisons of neutrophil dynamic parameters, including transit time, track length, velocity, and straightness across groups. The middle line represents the median value, and statistical significance was assessed using the Kruskal-Wallis test, followed by Dunn’s multiple-comparison test against the PBS group. *P < 0.05.

Next, we investigated the role of neutrophils in cisplatin-induced AKI, as neutrophil infiltration is a known key element in the disease (10, 21). As expected, after cisplatin induction, the count of *LysM*^gfp/+^ cells and Ly6G^+^ proportion in *LysM*^gfp/+^ cells significantly increased, which suggests an increase in neutrophils (Figure 4, A–C). Interestingly, 48 hours after cisplatin induction, *LysM*^gfp/+^ cell infiltration was initiated in the S2 segments in the proximal tubule, where cisplatin accumulation is known to occur (22–25) (Figure 4A). Although *LysM*^gfp/+^ cell infiltration was observed near the S2 segments in proximal tubules, neutrophil infiltration did not affect the peritubular microcirculation, as observed using fluorescence and cellular angiography (Figure 4D and Supplemental Video 6). While neutrophil blockade of capillaries was not evident, neutrophil depletion with an anti-Ly6G antibody injection was performed to further confirm the role of neutrophils in the cisplatin-induced AKI model. Unexpectedly, the Ly6G^+^ cell depletion model was significantly vulnerable to death, suggesting that systemic depletion of neutrophils may adversely affect the survival of the cisplatin-induced AKI model (23, 26, 27) (Figure 4E). The increased vulnerability was likely due to a reduction in white blood cell count, potentially leading to heightened susceptibility to infection (Supplemental Figure 5). To further investigate the role of neutrophils during proximal infiltration, we quantified multiple dynamic parameters (Figure 4F and Supplemental Video 7). Compared with the control (PBS) group, the transit time, representing the residence time of neutrophils within the field of view, was significantly prolonged in the cisplatin-induced AKI groups (Figure 4G). Similarly, track length and velocity were significantly increased in these groups (Figure 4, H and I). In contrast, the straightness index was significantly decreased, likely due to the increased presence of neutrophils with reduced motility near the tubule site (Figure 4J). Taken together, these findings indicate that neutrophils exhibited crawling dynamics rather than causing direct obstruction in the cisplatin-induced AKI.

### Role of CD11b in cisplatin-induced AKI.

**Figure 5:**
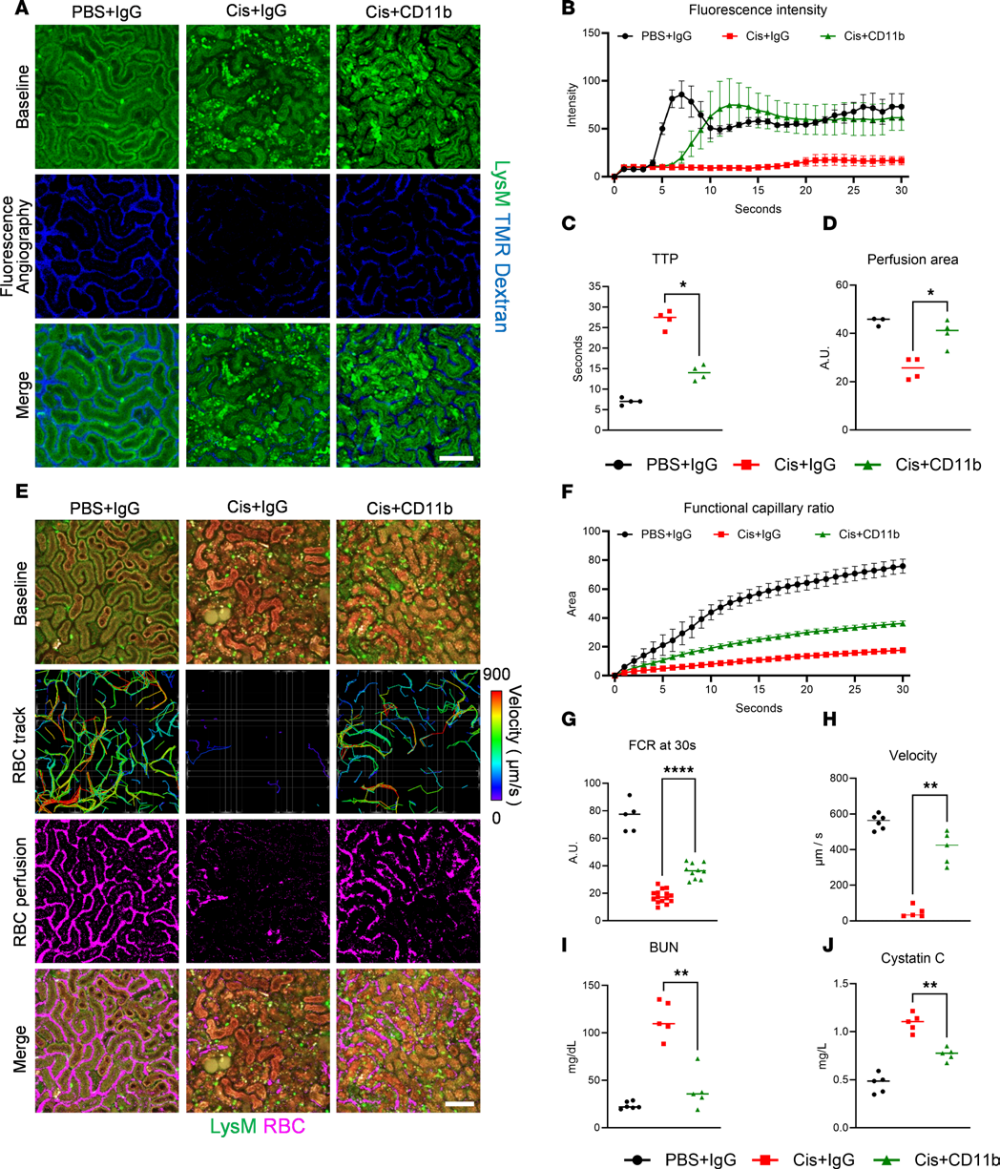
Role of anti-CD11b treatment in the cisplatin-induced AKI model. (**A**) Representative fluorescence angiography imaging in the PBS+IgG, Cis+IgG, and Cis+CD11b groups. See also Supplemental Video 8. Scale bar: 100 μm. (**B**) Time-intensity curve of fluorescence intensity in each group (*n* = 3 for each group). (**C** and **D**) Comparisons of time-to-peak (TTP) and perfusion area at 30 seconds between groups. (**E**) Representative cellular angiography imaging in the PBS+IgG, Cis+IgG, and Cis+CD11b groups. See also Supplemental Video 9. Scale bar: 100 μm. (**F**) Time-intensity curve of the functional capillary ratio (FCR) in each group (*n* = 5 for PBS, *n* = 10 for Cis 48, *n* = 15 for Cis 72). (**G** and **H**) Comparisons of FCR at 30 seconds and velocity between groups. (**I** and **J**) Comparisons of biomarkers (BUN and cystatin C) between groups. The middle line represents the median value, and statistical significance was assessed via the Mann-Whitney test (**C**, **D**, and **G**–**J**). *P < 0.05, **P < 0.01, ****P < 0.0001.

As an alternative strategy to neutrophil depletion, we next investigated the role of an anti-CD11b antibody in cisplatin-induced AKI, which is known to functionally deactivate neutrophils (28, 29). Using fluorescence angiography, anti-CD11b antibody treatment led to improvement of peritubular microcirculation, as revealed by decreased TTP (Figure 5, A–C, and Supplemental Video 8) and increased perfusion area (Figure 5D). Additionally, in cellular angiography, anti-CD11b antibody ameliorated disturbances in peritubular microcirculation, as indicated by both an increase in the FCR (Figure 5, E–G, and Supplemental Video 9) and the velocity of multiple erythrocytes (Figure 5, E and H). These findings were further validated by the plasma concentrations of BUN (Figure 5I) and cystatin C (Figure 5J). Notably, anti-CD11b antibody treatment did not reduce neutrophil accumulation (Supplemental Figure 6). Taken together, the evidence showed that anti-CD11b antibody treatment effectively improved the peritubular microcirculation in a cisplatin-induced AKI model.

## Discussion

In this study, we were able to visualize and quantify the peritubular microcirculation at the cellular level in a cisplatin-induced AKI model. Using 2 methods of angiography (fluorescence and cellular), we described how microcirculation is disturbed in a cisplatin-induced AKI model and derived variables representing the level of microcirculation. We found that the increase in TTP and the decrease in perfusion area in fluorescence angiography and the decrease in FCR in cellular angiography could be clearly identified before 72 hours, while the increase in current biomarkers in AKI (BUN and cystatin C) were not prominent until 96 hours after cisplatin induction. Furthermore, in the correlation graph, biomarkers were not discriminative in the early period, particularly at low concentrations. In contrast, the parameters related to the microcirculation (TTP, perfusion area, and FCR) were more sensitively distinguishable and reliable indicators of the progression toward renal failure in earlier stages. Overall, our study provides insight into the use of microcirculation variables as an early diagnostic tool for preclinical cisplatin-induced AKI models.

In our study, with fluorescence angiography, TTP was evaluated utilizing the temporal change in fluorescence intensity. The parameter was differentiated between the control and each period of the cisplatin-induced AKI group, indicating its potential as an imaging biomarker. Nevertheless, fluorescence angiography had limitations, as only a single opportunity was available during the contrast agent injection period, and additional measurements were unfeasible until washout, especially in the kidney, which works as an excretory organ. Additionally, longitudinal imaging of peritubular microcirculation in the same animal up to 96 hours was also technically unattainable due to the residual fluorescence in the tubule, particularly in models with reduced excretory function due to AKI. To address these limitations, we utilized cellular angiography, in which erythrocytes were fluorescently labeled, providing an opportunity to measure microcirculation without temporal limitations. The correlation of TTP with FCR was strong, suggesting that cellular angiography may be a substitute for fluorescence angiography. Furthermore, cellular angiography was able to capture the dynamics of thousands of individual erythrocytes, thereby providing detailed information on the pattern of microcirculation in a cisplatin-induced AKI model.

While it is generally recognized that peritubular capillaries branch from efferent arterioles derived from glomeruli and form a network near tubules, the distinction between segments of peritubular capillaries has not been extensively explored previously. In our study, where the role of the dynamics of fluorescence and cellular angiography were highlighted, we found that S1 segments of proximal peritubular capillaries were specifically vulnerable to cisplatin-induced AKI. Previous in vitro studies have shown that S1 cells are the most sensitive to cisplatin-induced cytotoxicity, whereas S3 cells accumulate cisplatin at the highest rate (30). The finding aligns with our observations, suggesting that the selective vulnerability of S1 peritubular capillaries may be linked to segment-specific cisplatin cytotoxicity. Furthermore, cisplatin is directly toxic to endothelial cells, and renal vasoconstriction caused by endothelial dysfunction and impaired vascular autoregulation is also a contributing factor in cisplatin-induced AKI (23). However, these mechanisms do not fully explain the specific vulnerability of the S1 segment. Initially, our hypothesis postulated that neutrophil infiltration in proximal tubules could potentially obstruct the proximal capillaries, thereby leading to interference with the peritubular microcirculation. However, our subsequent findings revealed that this hypothesis was incorrect. A possible hypothesis could be secretion of tubule-specific vasoactive factors that act on capillary pericytes and cause preferential capillary contractions (31). In addition, capillary obstruction may have been missed, or neutrophil-induced endothelial damages may have occurred at earlier time points, as suggested in other AKI models, such as tumor lysis syndrome–induced AKI (32) or ischemia-reperfusion injury (33). An alternative explanation is that there may exist an intermediate functional structure between the peritubular capillary in S1 and S2 segments of proximal tubules, which could potentially regulate blood flow and be affected by cisplatin. Further investigation is necessary to determine the precise factors and mechanisms contributing to the vulnerability of S1 segments of peritubular capillaries in cisplatin-induced AKI.

Consistent with previous studies, our study also indicates that neutrophil infiltration in the proximal tubule is increased during cisplatin-induced AKI. However, neutrophil depletion following cisplatin injection increased susceptibility to death, thereby limiting the opportunities for intravital imaging. One possible explanation may be that as cisplatin is a well-known suppressor of bone marrow, systemic side effects such as infection might have occurred during neutrophil depletion (23). In addition, neither neutrophil depletion using an anti–Gr-1 (RB6-8C5) antibody nor anti-Ly6G antibody exhibited beneficial effects in a cisplatin-induced AKI model (26, 34, 35). CD11b constitutes the Mac-1 complex with CD18, a key integrin that regulates neutrophil adhesion and migration in inflamed tissues, particularly in pulmonary capillaries (15, 29). While the anti-CD11b antibody has been previously recognized for its impact on neutrophil-mediated effects in various diseases, this study provides a perspective on the potential role of the anti-CD11b antibody in improving microcirculation in cisplatin-induced AKI (28, 36). Further studies are warranted to elucidate the underlying pathophysiological mechanisms regarding the role of anti-CD11b antibodies in cisplatin-induced AKI.

Previous studies have demonstrated visualization of peritubular microcirculation; however, they lacked comprehensive and extensive quantification (8). Furthermore, with recent technical advances in optics, biological sensors, and imaging windows, intravital imaging has been primarily focused on molecular and biological components, with less emphasis on microcirculation (14, 37). However, renal microcirculation is an integrative physiological component where complex cellular mechanisms, including inflammatory, oxidative, and nitrosative factors, contribute to the path leading to renal failure (38). Using fluorescence imaging and cellular angiography, we were able to demonstrate that peritubular microcirculation disturbance in cisplatin-induced AKI is initiated from the peritubular capillary of S1 segments, and neutrophil infiltration in the proximal tubule is not mechanically responsible for capillary obstruction.

In earlier studies, ultrasound or computed tomography imaging was utilized to identify microvascular structures with a resolution down to 30–40 micrometers (39–41). In this study, our imaging technique offered high-resolution capabilities of a few micrometers, allowing for the detection of individual erythrocytes and leukocytes, including their dynamics, which is not achievable with ultrasound or computed tomography. While the penetration depth and invasiveness are inherent technical challenges, ongoing advancements in endoscopic devices (42) and laser sources (43) can help to address these limitations.

In conclusion, using intravital microscopy, we achieved direct visualization of the peritubular microcirculation in cisplatin-induced AKI. With fluorescence and cellular angiography, multifaceted quantification with distinguishing the status of microcirculation in each stage was feasible. Our findings highlight that functional inactivation of neutrophils using the anti-CD11b antibody may serve as a potential therapeutic strategy for restoring peritubular microcirculation in cisplatin-induced AKI. Moreover, integrating microcirculation analysis with functional imaging of nearby individual cells may assist in understanding their role and further provide insights into the pathophysiology of various renal diseases. This imaging system has the potential to become a useful tool for investigating renal diseases that affect peritubular microcirculation and for evaluating potential therapeutic candidates for various renal diseases in a preclinical setting.

## Methods

### Sex as a biological variable.

Due to the protective effects of estrogen in females and the higher clearance rate of cisplatin in males, there are known sex-specific differences in cisplatin-induced nephrotoxicity (44). To minimize variability, our study exclusively examined male mice. It is unknown whether the findings are relevant for female mice.

### Study design.

Mice were randomly assigned to either time after treatment with cisplatin or PBS (*n* = 3 to 6 per group). For the allocation of each group, randomization with online random number generators was performed before either antibody injection by the first investigator. A second investigator unaware of the allocation performed intravital imaging and measurement order was randomized with online random number generators.

### Mice and animal models.

Twelve-week-old male mice (C57BL/6N mice (OrientBio) and *LysM*^gfp/+^ (bred in the SNUBH animal facility) weighing 25–30 g were utilized in this study. To generate a cisplatin-induced AKI model, a high dose of cisplatin (20 mg/kg) (Dong-A ST Inc) was intraperitoneally administered (5). The median survival in this model was 146.5 hours. In the control group, the same volume of PBS was injected into the peritoneum. For the neutrophil depletion model (Ly6G dep), 200 μg of anti-Ly6G monoclonal antibody (clone 1A8, 551459, BD Biosciences) was intraperitoneally injected 24 hours prior to modeling (15). In the CD11b depletion model (Cis-CD11b), 100 μg of anti-CD11b monoclonal antibody (clone M1/70, 553307, BD Biosciences) was intraperitoneally injected 24 hours after cisplatin injection (15). The control antibody (IgG, 553985, BD Biosciences) was intraperitoneally injected into the other groups (PBS+IgG and Cis+IgG).

### Fluorescent dye and antibody in intravital imaging.

To visualize the vessel with a fluorescent dye, FITC-dextran (2,000 kDa, FD2000S, Merck) or TMR-dextran (2,000 kDa, D7139, Thermo Fisher Scientific; 4 kDa, T1037, Merck) was injected via a vascular catheter during imaging (45, 46). Erythrocytes were obtained by cardiac puncture from anesthetized donor mice and then labeled with Vybrant DiD (V22887, Thermo Fisher Scientific) (15). Fifty million counts of DiD-labeled erythrocytes were injected via a vascular catheter during imaging (47). To label the vessels, 25 μg of anti-CD31 monoclonal antibody (clone 390, 553708, BD Bioscience) conjugated with the fluorophore Alexa Fluor 647 (A-20006, Thermo Fisher Scientific) was injected via the tail vein 2 hours before imaging (48). To label the S2 segments in proximal tubules, 25 μg of anti-OAT1 antibody (clone SCL22A6, PA5-102488, Thermo Fisher Scientific) conjugated with the fluorophore Alexa Fluor 647 was injected via the tail vein 24 hours before imaging. To specifically label neutrophils in vivo, 25 μg of anti-Ly6G monoclonal antibody (clone 1A8, 551459, BD Bioscience) conjugated with the fluorophore Alexa Fluor 647 was injected via the tail vein 2 hours before imaging (15).

### Intravital imaging system.

To visualize peritubular microcirculation in the kidney, video-rate laser scanning confocal microscopy (IVIM-C, IVIM Technology) was utilized. Additional details of the imaging system are provided elsewhere (15, 45). Briefly, the system utilized 3 continuous laser modules (wavelengths at 488, 561, 640 nm) as excitation light sources, while 2 axes of scanning were facilitated using a rotating polygonal mirror (*x*) and galvanometer scanning mirror (*y*). The system employed 4 objective lenses (UPLSAP0, 4×, NA 0.16, Nikon; UYplanSApo, 10×, NA 0.4, Nikon; PlanApoλ, 20×, NA 0.75, Nikon; and Olympus, LUCPLFLN, 40×, NA 0.6), and the detector consisted of photomultiplier tubes through bandpass filters with pass bands of 500–550 nm, 582–618 nm, and 663–733 nm.

### Intravital kidney imaging.

All experimental mice were anesthetized using a combination of zolazepam/tiletamine (Zoletil, 20 mg/kg; Virbac) and xylazine (Rompun, 12 mg/kg; Bayer). Rectal temperature was monitored using a thermal probe connected to a feedback-regulated heating pad (RightTemp, Kent Scientific), which maintained the body temperature at 37.0°C. To enable intravenous injection of molecular dyes and cells, a vascular catheter was assembled using a 30-gauge needle (Omnican 50, B. Braun) and polyethylene tubing (PE10, 427401, BD Biosciences) and catheterized into the tail vein (49). Vascular patency was verified by saline injection. The mice were positioned in the right lateral decubitus position, and an incision was made in the left flank. After careful dissection of the skin, muscle, and peritoneum, the left kidney was exposed without direct contact by cautiously compressing the abdomen to minimize respiratory and other motion artifacts. Finally, a slide glass was attached with as little pressure as possible to the exposed kidney for intravital imaging (48).

### Image processing.

The imaging system was configured to capture images at a rate of 30 frames per second, with each frame consisting of 512 × 512 pixels. Real-time image frames were averaged over 30 frames to improve contrast and signal-to-noise ratio using a custom-made (MATLAB, Mathworks) code (15). Prior to averaging, each frame was processed with an image registration algorithm to minimize motion artifacts (15, 45).

The intensity of fluorescent dextran and perfusion area were evaluated using ImageJ (https://imagej.nih.gov/ij/). TTP is the time to the apex of the time-intensity curve, which reflects the time it takes until the fluorescent dextran bolus reaches the tissue. Functional capillary imaging analysis was performed using a real-time video of DiD-labeled erythrocytes flowing in capillaries (15). After splitting the colors of the video, the sequential images of the channel detecting DiD were processed using a median filter with a radius of 2 pixels to enhance the signal-to-noise ratio. A maximal intensity projection of 900 frames (30 seconds) was then conducted to visualize the functional capillary perfused by erythrocytes. To eliminate noise from autofluorescence in the tubule, the image with maximal intensity projection was subtracted from the baseline averaged imaging. The FCR was determined by calculating the ratio of the functional capillary area (DiD-labeled RBCs) to the total capillary area (vessel area detected by dextran signaling). The calculation of FCR, including the image processing described above, was performed using ImageJ. Track analysis of erythrocytes and neutrophils and plotting tracks were performed using the autoregressive method in IMARIS 9.0 (15) (Oxford Instruments).

### Biomarker measurement.

To assess the severity of AKI with current clinical biomarkers, blood samples of 500 μL were collected by cardiac puncture under deep intraperitoneal anesthesia following intravital imaging. After isolation of plasma by centrifugation, BUN levels were measured using a colorimetric assay (EIABUN, Thermo Fisher Scientific), and cystatin C levels were measured using an enzyme-linked immunosorbent assay (MSCTC0, R&D Systems). Animals were euthanized with a CO_2_ chamber immediately after blood sampling.

### Statistics.

Data are presented as the median to represent values of the group. Statistical differences between medians were determined by Mann-Whitney test or Kruskal-Wallis test followed by Dunn’s multiple-comparison test, as appropriate. Samples were excluded from the analysis if sampling failure occurred or if early mortality prevented data collection. A *P* value of less than 0.05 was consider significant, and all analyses were performed using Prism 9.0 (GraphPad Software).

### Study approval.

All animal experiments were approved by the Institutional Animal Care and Use Committee (IACUC) of SNUBH (protocol no. BA-1910-281-079), and animals were housed in a facility accredited by AAALAC International (no. 001847) in accordance with the NIH *Guide for the Care and Use of Laboratory Animals* (National Academies Press, 2011).

### Data availability.

Supporting numerical data values presented in the graphs are provided as a Supporting Data Values file. Raw image files and videos generated by intravital microcopy are available upon request from the corresponding author.

## Author contributions

IP and YHJ designed the research study. IP and Seonghye Kim conducted experiments with the help of YWU and HEK. IP and Seonghye Kim acquired data and performed data analysis. IP and PK contributed to new methods. IP wrote the original draft of the manuscript under the guidance and revision of JHL, Sejoong Kim, PK, and YHJ. IP, JHL, Sejoong Kim, PK, and YHJ edited the manuscript. All authors reviewed and edited the manuscript.

## Supplementary Material

Supplemental data

Supplemental video 1

Supplemental video 2

Supplemental video 3

Supplemental video 4

Supplemental video 5

Supplemental video 6

Supplemental video 7

Supplemental video 8

Supplemental video 9

Supporting data values

## Version 1 04/08/2025

In-Press Preview

## Version 2 05/08/2025

Electronic publication

## References

[1] Pabla N, Dong Z (2008). Cisplatin nephrotoxicity: mechanisms and renoprotective strategies. Kidney Int.

[2] Dasari S, Tchounwou PB (2014). Cisplatin in cancer therapy: molecular mechanisms of action. Eur J Pharmacol.

[3] Yao X (2007). Cisplatin nephrotoxicity: a review. Am J Med Sci.

[4] Periyasamy-Thandavan S (2008). Autophagy is cytoprotective during cisplatin injury of renal proximal tubular cells. Kidney Int.

[5] Xu Y (2015). A role for tubular necroptosis in cisplatin-induced AKI. J Am Soc Nephrol.

[6] Sears SM, Siskind LJ (2021). Potential therapeutic targets for cisplatin-induced kidney injury: lessons from other models of AKI and fibrosis. J Am Soc Nephrol.

[7] Sun N (2021). Development of a photoacoustic microscopy technique to assess peritubular capillary function and oxygen metabolism in the mouse kidney. Kidney Int.

[8] Yamamoto T (2002). Intravital videomicroscopy of peritubular capillaries in renal ischemia. Am J Physiol Renal Physiol.

[9] Kramann R (2014). Fluorescence microangiography for quantitative assessment of peritubular capillary changes after AKI in mice. J Am Soc Nephrol.

[10] Deng B (2017). The leukotriene B_4_-leukotriene B_4_ receptor axis promotes cisplatin-induced acute kidney injury by modulating neutrophil recruitment. Kidney Int.

[11] Miller RP (2010). Mechanisms of cisplatin nephrotoxicity. Toxins (Basel).

[12] Guven G (2020). Microcirculation: physiology, pathophysiology, and clinical application. Blood Purif.

[13] Pittet MJ, Weissleder R (2011). Intravital imaging. Cell.

[14] Martins JR (2021). Intravital kidney microscopy: entering a new era. Kidney Int.

[15] Park I (2019). Neutrophils disturb pulmonary microcirculation in sepsis-induced acute lung injury. Eur Respir J.

[16] Hato T (2018). Kidney imaging: intravital microscopy. Methods Mol Biol.

[17] Nakano D (2015). Reduction of tubular flow rate as a mechanism of oliguria in the early phase of endotoxemia revealed by intravital imaging. J Am Soc Nephrol.

[18] Kalakeche R (2011). Endotoxin uptake by S1 proximal tubular segment causes oxidative stress in the downstream S2 segment. J Am Soc Nephrol.

[19] Hwang JS (2010). Expression of OAT1 and OAT3 in differentiating proximal tubules of the mouse kidney. Histol Histopathol.

[20] Lungkaphin A (2006). Relative contribution of OAT1 and OAT3 transport activities in isolated perfused rabbit renal proximal tubules. Biochim Biophys Acta.

[21] Tadagavadi R, Reeves WB (2017). Neutrophils in cisplatin AKI-mediator or marker?. Kidney Int.

[22] Faubel S (2004). Caspase-1-deficient mice are protected against cisplatin-induced apoptosis and acute tubular necrosis. Kidney Int.

[23] Ozkok A, Edelstein CL (2014). Pathophysiology of cisplatin-induced acute kidney injury. Biomed Res Int.

[24] Hall AM (2013). In vivo multiphoton imaging of mitochondrial structure and function during acute kidney injury. Kidney Int.

[25] Hukriede NA (2022). Experimental models of acute kidney injury for translational research. Nat Rev Nephrol.

[26] Faubel S (2007). Cisplatin-induced acute renal failure is associated with an increase in the cytokines interleukin (IL)-1beta, IL-18, IL-6, and neutrophil infiltration in the kidney. J Pharmacol Exp Ther.

[27] Jang HR, Rabb H (2015). Immune cells in experimental acute kidney injury. Nat Rev Nephrol.

[28] Jaeschke H (1993). Functional inactivation of neutrophils with a Mac-1 (CD11b/CD18) monoclonal antibody protects against ischemia-reperfusion injury in rat liver. Hepatology.

[29] Yipp BG (2017). The lung is a host defense niche for immediate neutrophil-mediated vascular protection. Sci Immunol.

[30] Kroning R (1999). Differential effects of cisplatin in proximal and distal renal tubule epithelial cell lines. Br J Cancer.

[31] Hall CN (2014). Capillary pericytes regulate cerebral blood flow in health and disease. Nature.

[32] Arnaud M (2022). Tumor lysis syndrome and AKI: beyond crystal mechanisms. J Am Soc Nephrol.

[33] Awad AS (2009). Compartmentalization of neutrophils in the kidney and lung following acute ischemic kidney injury. Kidney Int.

[34] Tadagavadi RK (2015). Dendritic cell protection from cisplatin nephrotoxicity is independent of neutrophils. Toxins (Basel).

[35] Chan AJ (2014). Innate IL-17A-producing leukocytes promote acute kidney injury via inflammasome and Toll-like receptor activation. Am J Pathol.

[36] Ahn GO (2010). Inhibition of Mac-1 (CD11b/CD18) enhances tumor response to radiation by reducing myeloid cell recruitment. Proc Natl Acad Sci U S A.

[37] Peti-Peterdi J (2016). Intravital imaging in the kidney. Curr Opin Nephrol Hypertens.

[38] Ince C (2014). The central role of renal microcirculatory dysfunction in the pathogenesis of acute kidney injury. Nephron Clin Pract.

[39] Chen Q (2020). Ultrasound super-resolution imaging provides a noninvasive assessment of renal microvasculature changes during mouse acute kidney injury. Kidney Int.

[40] Missbach-Guentner J (2018). 3D virtual histology of murine kidneys -high resolution visualization of pathological alterations by micro computed tomography. Sci Rep.

[41] Apelt K (2021). Imaging the renal microcirculation in cell therapy. Cells.

[42] Kim P (2010). In vivo wide-area cellular imaging by side-view endomicroscopy. Nat Methods.

[43] Choe K (2022). Intravital three-photon microscopy allows visualization over the entire depth of mouse lymph nodes. Nat Immunol.

[44] Eshraghi-Jazi F, Nematbakhsh M (2022). Sex difference in cisplatin-induced nephrotoxicity: laboratory and clinical findings. J Toxicol.

[45] Park I (2018). Intravital imaging of a pulmonary endothelial surface layer in a murine sepsis model. Biomed Opt Express.

[46] Hwang Y (2017). In vivo cellular-level real-time pharmacokinetic imaging of free-form and liposomal indocyanine green in liver. Biomed Opt Express.

[47] Seo H (2015). In vivo quantitation of injected circulating tumor cells from great saphenous vein based on video-rate confocal microscopy. Biomed Opt Express.

[48] Lee EM (2018). Effect of resveratrol treatment on graft revascularization after islet transplantation in streptozotocin-induced diabetic mice. Islets.

[49] Park I, Kim P (2021). Stabilized longitudinal in vivo cellular-level visualization of the pancreas in a murine model with a pancreatic intravital imaging window. J Vis Exp.

