# Supplementary material for: Intravital imaging of peritubular microcirculation impairment in cisplatin-induced acute kidney injury
Source: JCI Insight. 2025 Apr 8;10(9):e178689. doi: 10.1172/jci.insight.178689 (PMC12128961; doi:10.1172/jci.insight.178689)
Supplement: Supplemental data [file jciinsight-10-178689-s006.pdf]

Supplemental materials for

Intravital imaging of peritubular microcirculation impairment in cisplatin-induced acute kidney injury

Inwon Park, Seonghye Kim, Young Woo Um, Hee Eun Kim, Jae Hyuk Lee, Sejoong Kim, Pilhan Kim, You Hwan Jo*

*Corresponding authors. E-mail: emdryhj@gmail.com

This material includes the following:

Supplemental Figure 1. Representative fluorescence angiography using dextran with different molecular weight
Supplemental Figure 2. Representative fluorescence angiography confirming the preservation of peritubular microcirculation around S2 segments
Supplemental Figure 3. Tubular area changes following cisplatin administration
Supplemental Figure 4. Intravital imaging of erythrocyte velocity in ear artery
Supplemental Figure 5. White blood cell count and renal biomarkers in the Ly6G depletion model in cisplatin-induced AKI
Supplemental Figure 6. LysM+ cell count in anti-CD11b treatment model in cisplatin-induced AKI
Supplemental Video 1. Fluorescence angiography of peritubular microcirculation in cisplatin-induced AKI
Supplemental Video 2. Fluorescence angiography using two different size of molecular weight of peritubular microcirculation in cisplatin-induced AKI
Supplemental Video 3. Fluorescence angiography using OAT1 antibody to confirm S2 segments of proximal tubule
Supplemental Video 4. Cellular angiography of peritubular microcirculation in cisplatin-induced AKI
Supplemental Video 5. Mapping of the functional capillary ratio using cellular angiography in peritubular microcirculation in cisplatin-induced AKI
Supplemental Video 6. Neutrophil infiltration does not impede surrounding microcirculation in cisplatin-induced AKI
Supplemental Video 7. Neutrophil dynamics in proximal tubules in cisplatin-induced AKI
Supplemental Video 8. Fluorescence angiography of peritubular microcirculation after CD11b antibody treatment for cisplatin-induced AKI
Supplemental Video 9. Cellular angiography of peritubular microcirculation after CD11b antibody treatment for cisplatin-induced AKI

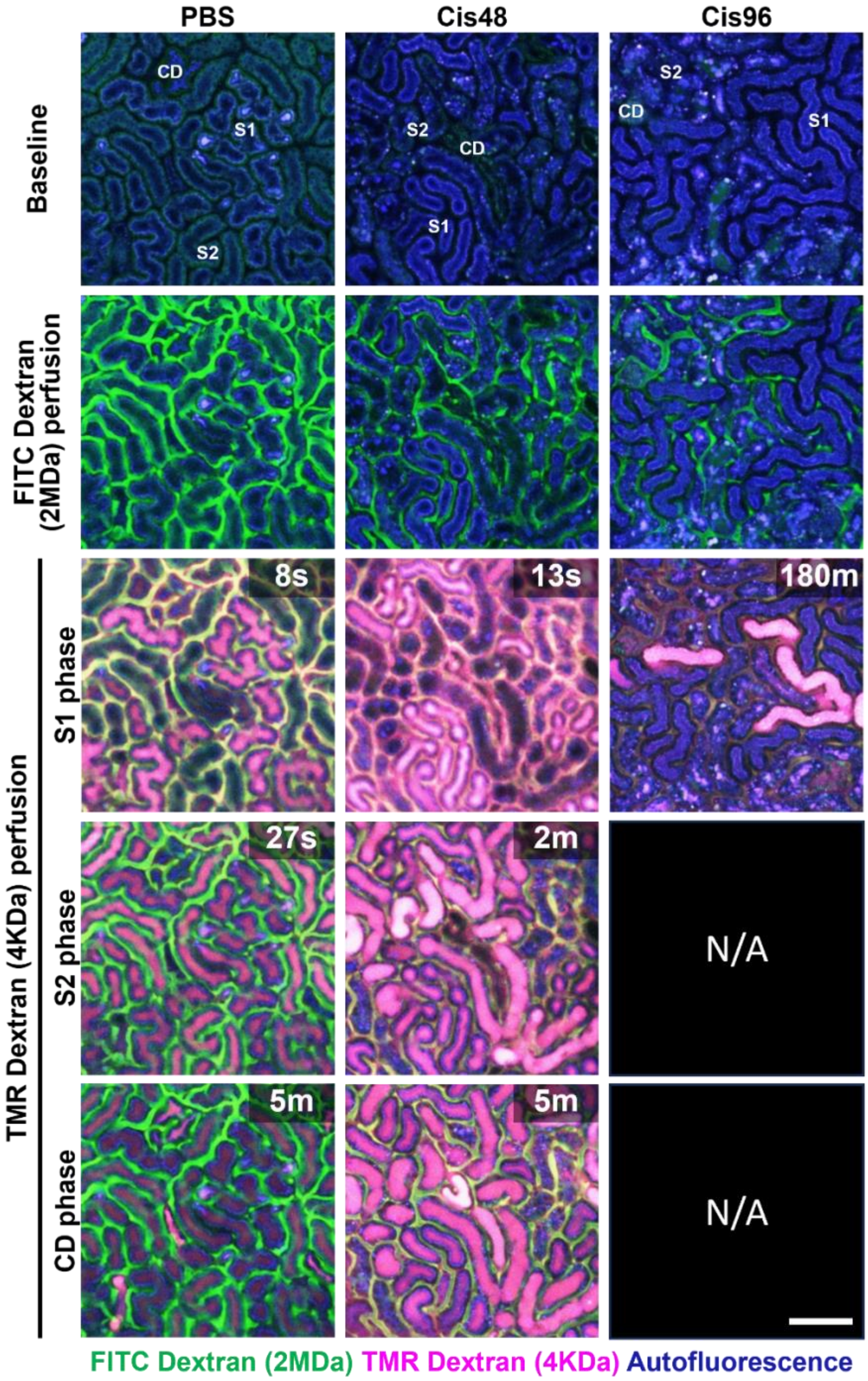

Supplemental Figure 1. Representative fluorescence angiography using dextran with different molecular weight

Representative fluorescence angiography with FITC Dextran (2MDa, Green) and TMR Dextran (4kDa, Red) imaging in the PBS, Cis48, and Cis96 groups. Proximal tubules, including S1 and S2, and distal tubules, including collecting ducts, can be easily distinguished through sequential phases. See also Supplemental Video 2. The time after TMR perfusion is indicated in upper right corner. The blue color represents autofluorescence. Scale bar, 100 μm. S1, S1 segments of proximal tubule; S2, S2 segments of proximal tubule; CD, Collecting duct; N/A, not applicable.

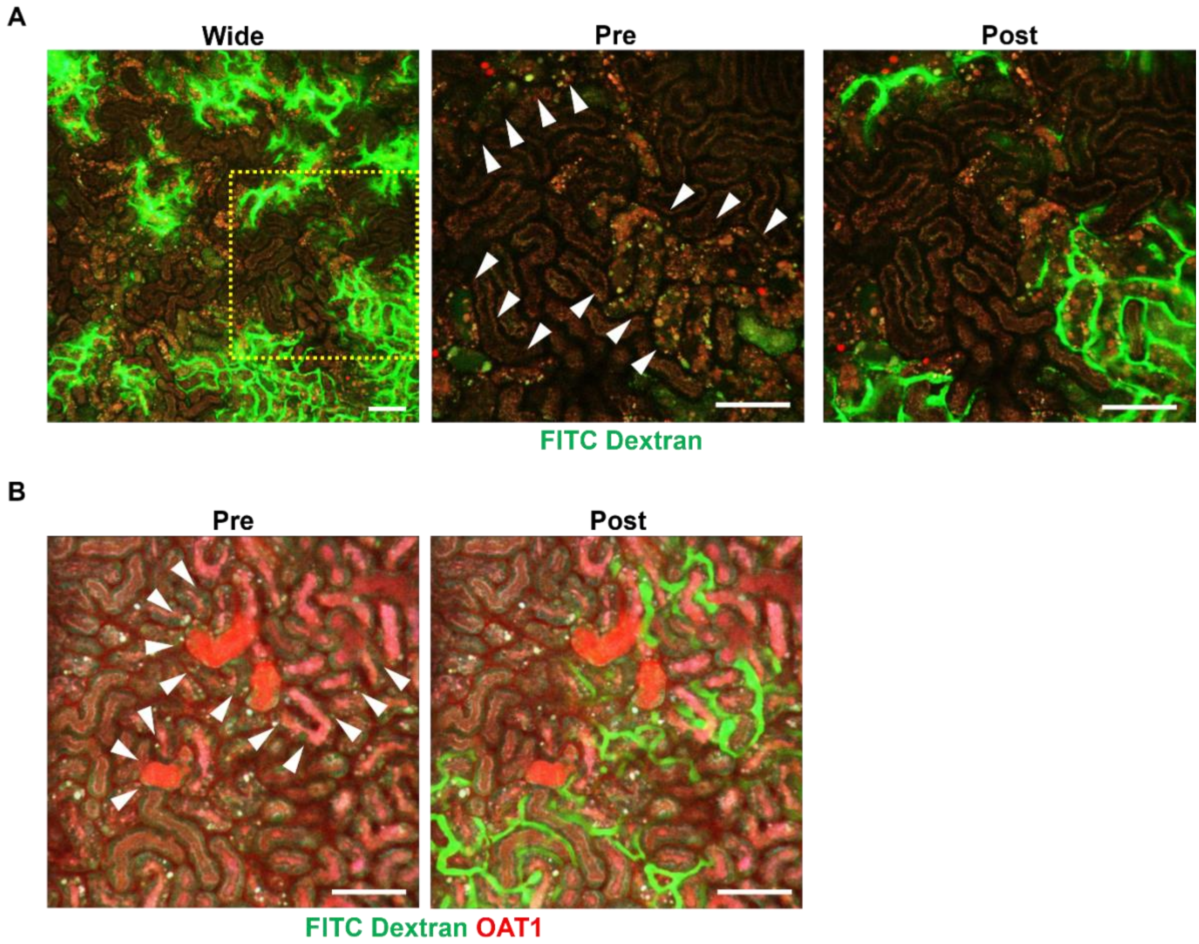

Supplemental Figure 2. Representative fluorescence angiography confirming the preservation of peritubular microcirculation around S2 segments

(**A**) The yellow dotted rectangle indicates the magnified field in the right panel. Peritubular capillaries surrounding tubules with high autofluorescence signals, presumed to be S2 segments of proximal tubules (as referenced in Supplemental Fig 1. and Supplemental Video 2), are perfused with FITC-dextran (green). In contrast, tubules without autofluorescence signals, corresponding to S1 segments of proximal tubules, are not perfused with FITC-dextran. (**B**) Immunostaining with the OAT1 antibody (red) highlights the localization of organic anion transporter 1 (OAT1) in S2 segments of proximal tubules. The fluorescence signals of FITC-dextran (green) perfusing the capillary further confirm the association between capillary perfusion and tubular segment identity. See also Supplemental Video 3. Scale bar, 100 μm.

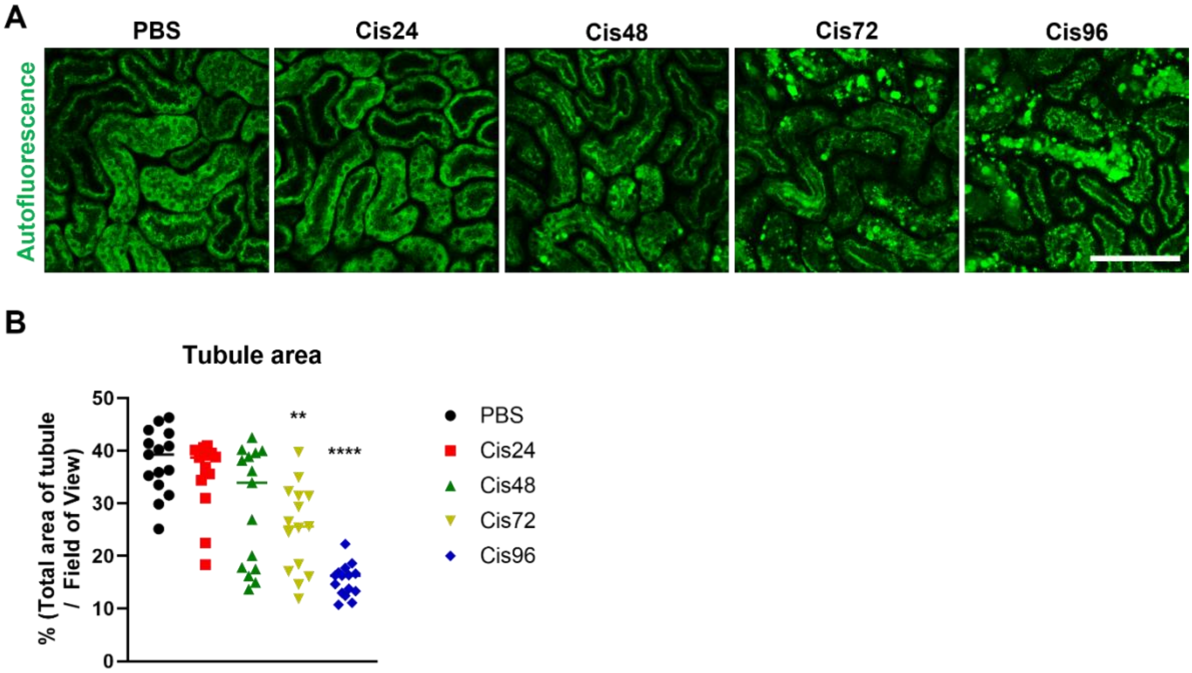

Supplemental Figure 3. Tubular area changes following cisplatin administration

(**A**) Representative fluorescence images of proximal tubules at different time points following cisplatin administration. Scale bar, 100 µm. (**B**) Quantification of tubular area shows a progressive decrease starting 72 hours post-cisplatin administration. Data suggest that increase of tubular area such as swelling may not be the primary contributor to peritubular microcirculation impairment. The middle line represents the median value, and statistical significance was assessed using the Kruskal-Wallis test, followed by Dunn's multiple comparisons test against the PBS group. * denotes comparison with the PBS group; *: P <0.05, ****: P < 0.0001.

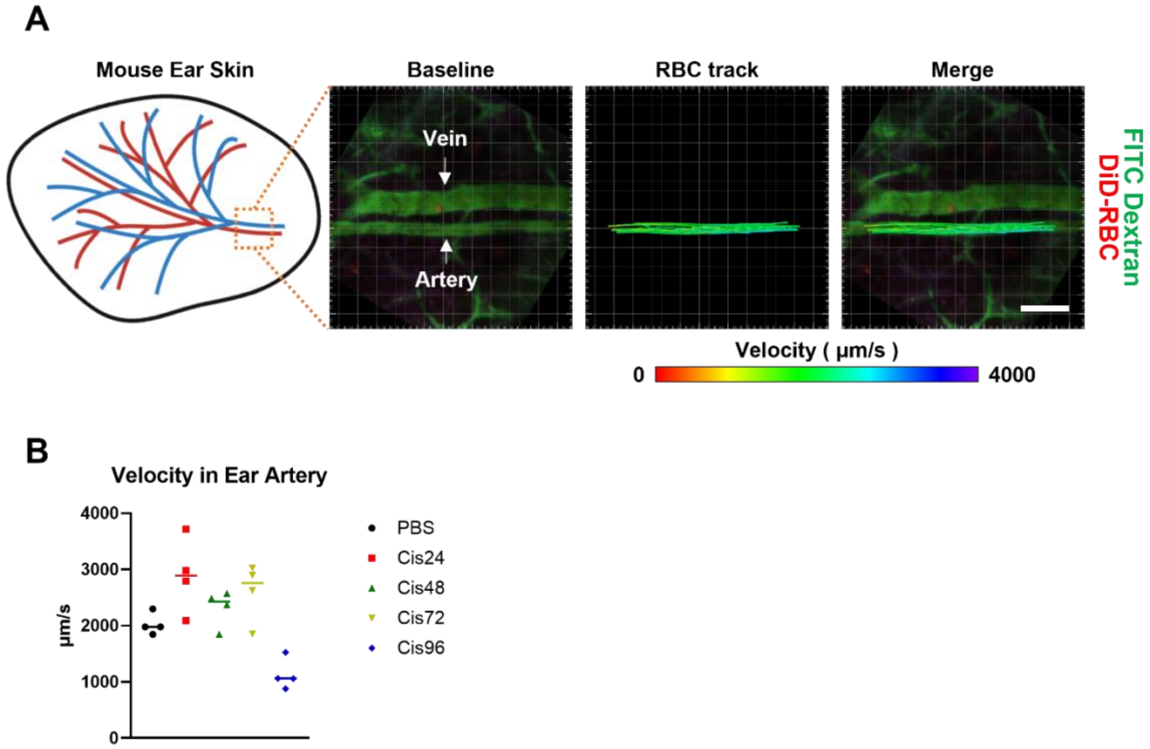

Supplemental Figure 4. Intravital imaging of erythrocyte velocity in ear artery

To evaluate the systemic perfusion in the cisplatin-induced AKI model, intravital imaging was additionally performed on the ear vasculature. (**A**) The most proximal artery in the ear was selected for analysis, as illustrated in schematic. Representative fluorescence images show FITC-dextran labeled plasma flow (green), DiD-labeled RBC, and color-coded velocity of RBC track. (**B**) Erythrocyte velocity was analyzed by tracking more than 30 erythrocytes per field in the ear artery. The mean velocity for each animal is represented as a dot in the graph. Until 96 hours after cisplatin injection, the mean velocity of erythrocyte in the ear artery was not decreased. The middle line represents the median value, and statistical significance was assessed using the Kruskal-Wallis test, followed by Dunn’s multiple comparisons test against the PBS group. * denotes comparison with the PBS group., *: P <0.05. Scale bar, 100 μm.

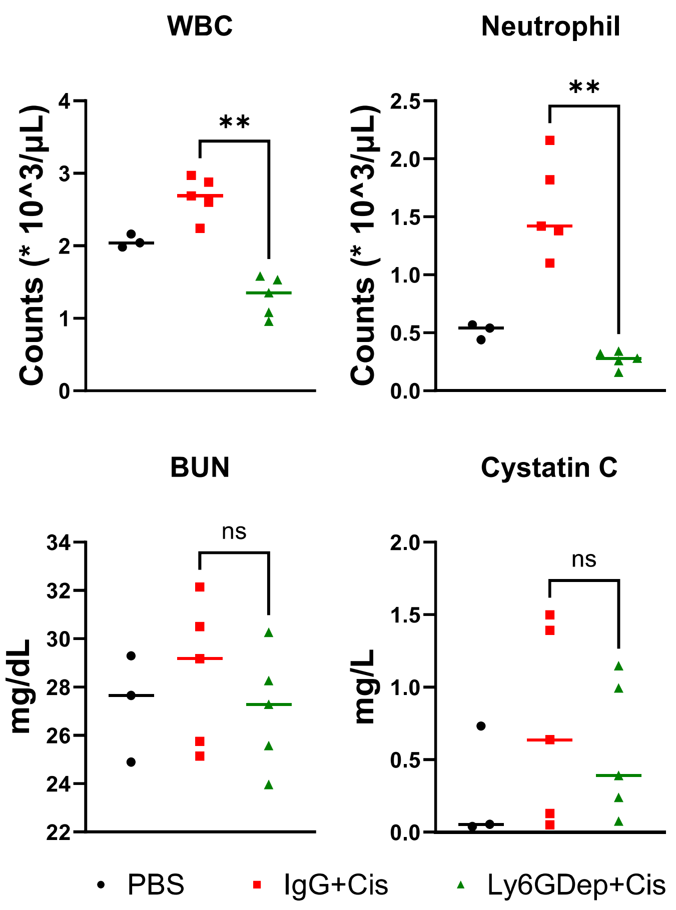

Supplemental Figure 5. White blood cell count and renal biomarkers in the Ly6G depletion model in cisplatin-induced AKI

Comparisons of white blood cell (WBC), neutrophil, BUN, and Cystatin C at 48 hours post-cisplatin period in Ly6G depletion model of cisplatin-induced AKI. To investigate the cause of early mortality in Ly6G-depleted mouse, WBC, neutrophils, BUN, and Cystatin C levels were evaluated. While no significant difference was identified in BUN and Cystatin C, WBC and neutrophil count was significantly reduced, suggesting increased susceptibility to infection as a contributing factor to mortality. The middle line represents the median value, and statistical significance was assessed via Mann–Whitney test. * denotes comparison with the groups., **: *P* <0.01., ns: non-significant.

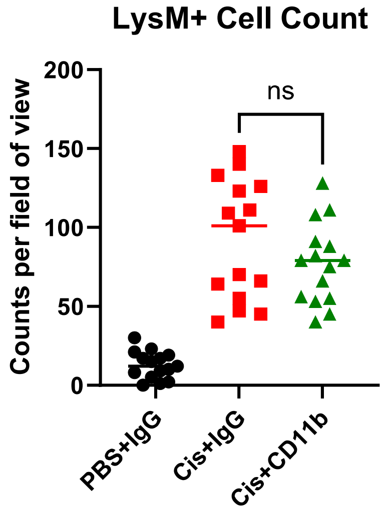

Supplemental Figure 6. LysM+ cell count in anti-CD11b treatment model in cisplatin-induced AKI

Comparison of LysM+ cell count per field of view in the anti-CD11b treatment model of cisplatin-induced AKI. The middle line represents the median value, and statistical significance was assessed via Mann–Whitney test. ns: non-significant.

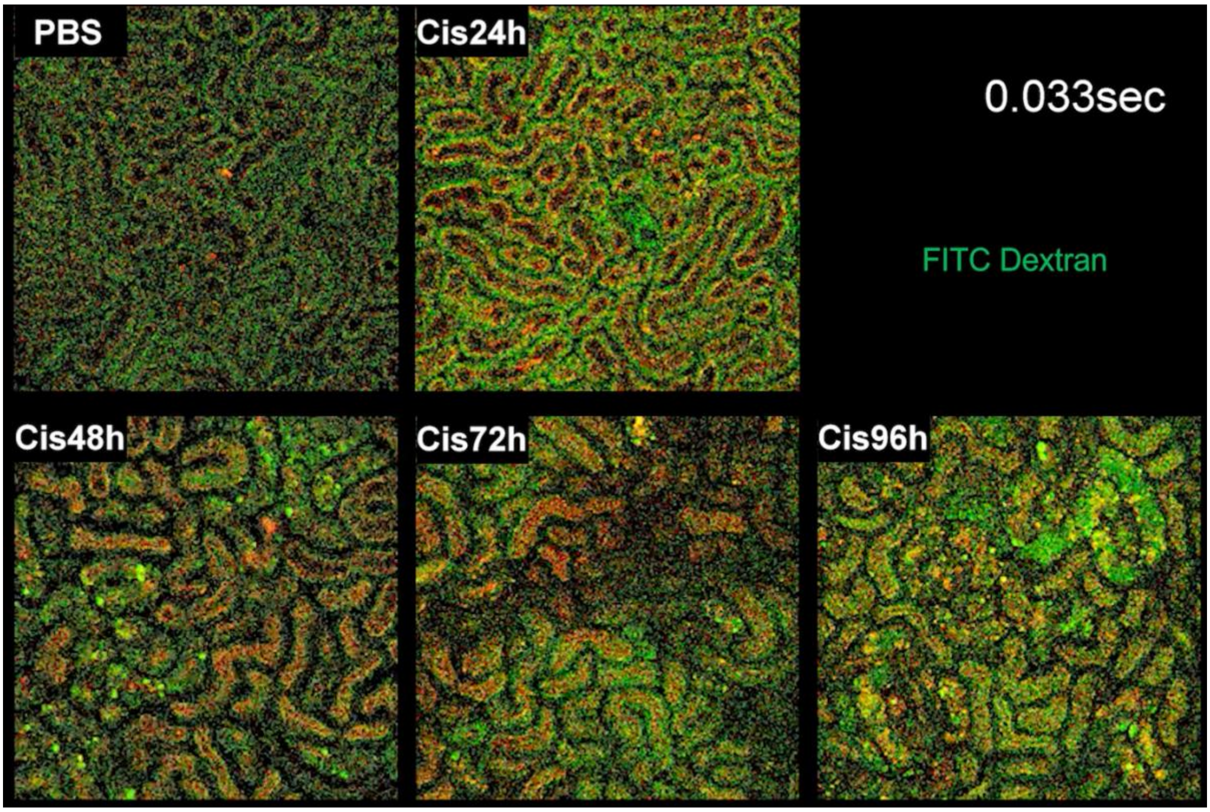

Supplemental Video 1. Fluorescence angiography of peritubular microcirculation in cisplatin-induced AKI

FITC–dextran (green) was intravenously injected into C57BL/6N mice via a tail vein catheter. The perfusion of peritubular capillaries progressively decreased in the group with cisplatin-induced AKI compared to the control group (PBS). This video corresponds to Figure 1A. Time is marked as SS.SSS (seconds).

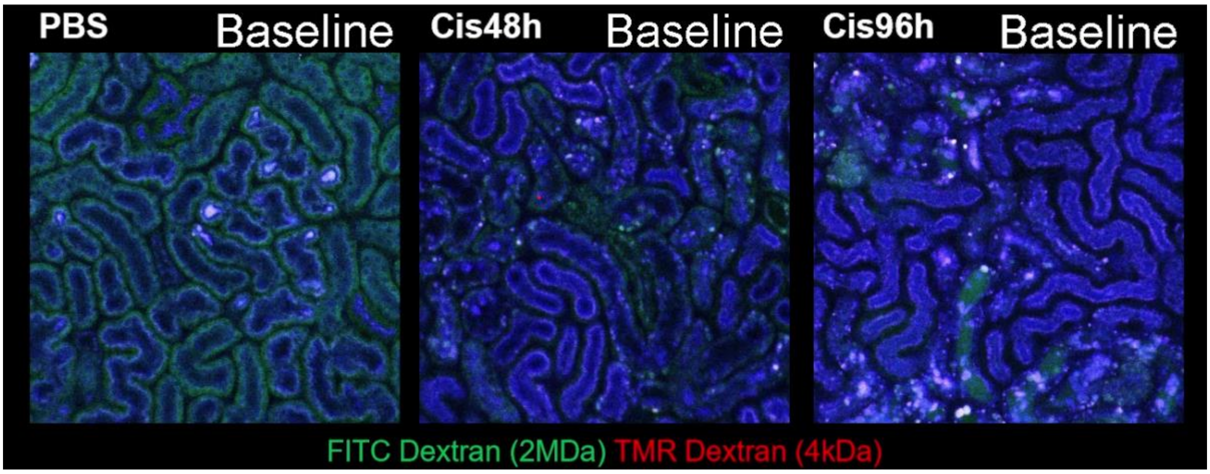

Supplemental Video 2. Fluorescence angiography using two different size of molecular weight of peritubular microcirculation in cisplatin-induced AKI

A high molecular weight (2MDa) of FITC–dextran (green) was intravenously injected into C57BL/6N mice via a tail vein catheter. Subsequently, a low molecular weight (4KDa) of TMR-dextran (red) was intravenously administered to distinguish each segment of tubule. Each phase (S1, S2, CD) of the models were facilitated to discerned through sequences. Using this method, it was determined that tubules exhibiting strong autofluorescence signals corresponded to the S2 segments of proximal tubules. This video corresponds to Supplemental Figure 1. Time is marked as HH:MM:SS (hours, minutes, seconds).

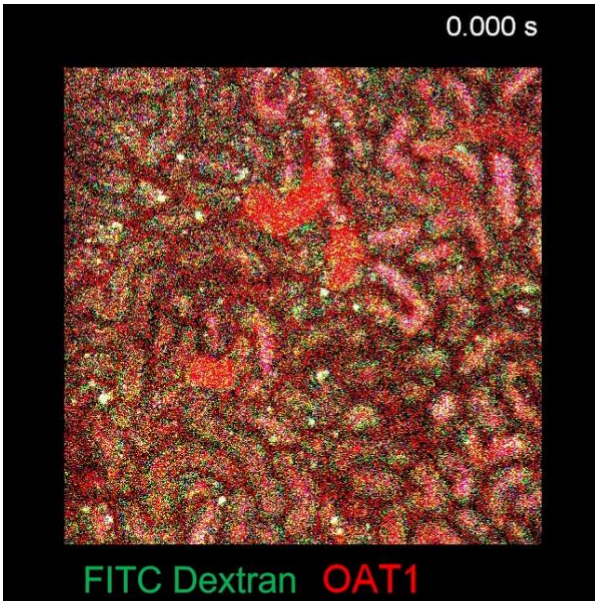

Supplemental Video 3. Fluorescence angiography using OAT1 antibody to confirm S2 segments of proximal tubule

An OAT1 antibody conjugated with Alexa647 was injected 24 hours before imaging, and high molecular weight (2MDa) FITC–dextran (green) was intravenously injected into C57BL/6N mice via a tail vein catheter. Capillary perfusion around tubules stained with the OAT1 antibody, presumed to be S2 segments of the proximal tubule, was preserved. This video corresponds to Supplemental Figure 2B. Time is marked as SS.SSS (seconds).

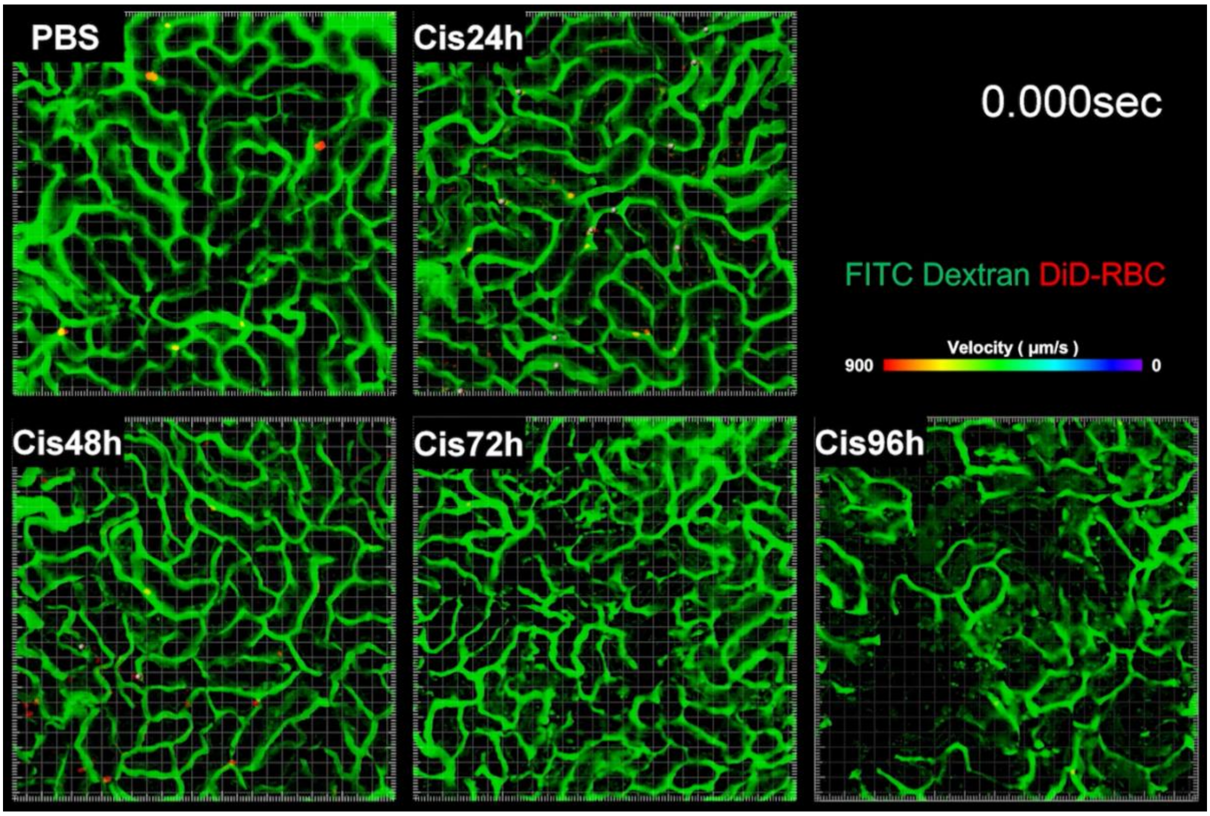

Supplemental Video 4. Cellular angiography of peritubular microcirculation in cisplatin-induced AKI

FITC–dextran (green) and DiD-labeled erythrocytes (red) were intravenously injected into C57BL/6N mice via a tail vein catheter. The color bar represents the mean velocity of the erythrocyte track (0-900 μm/sec). This video corresponds to Figure 2A. Time is marked as SS.SSS (seconds).

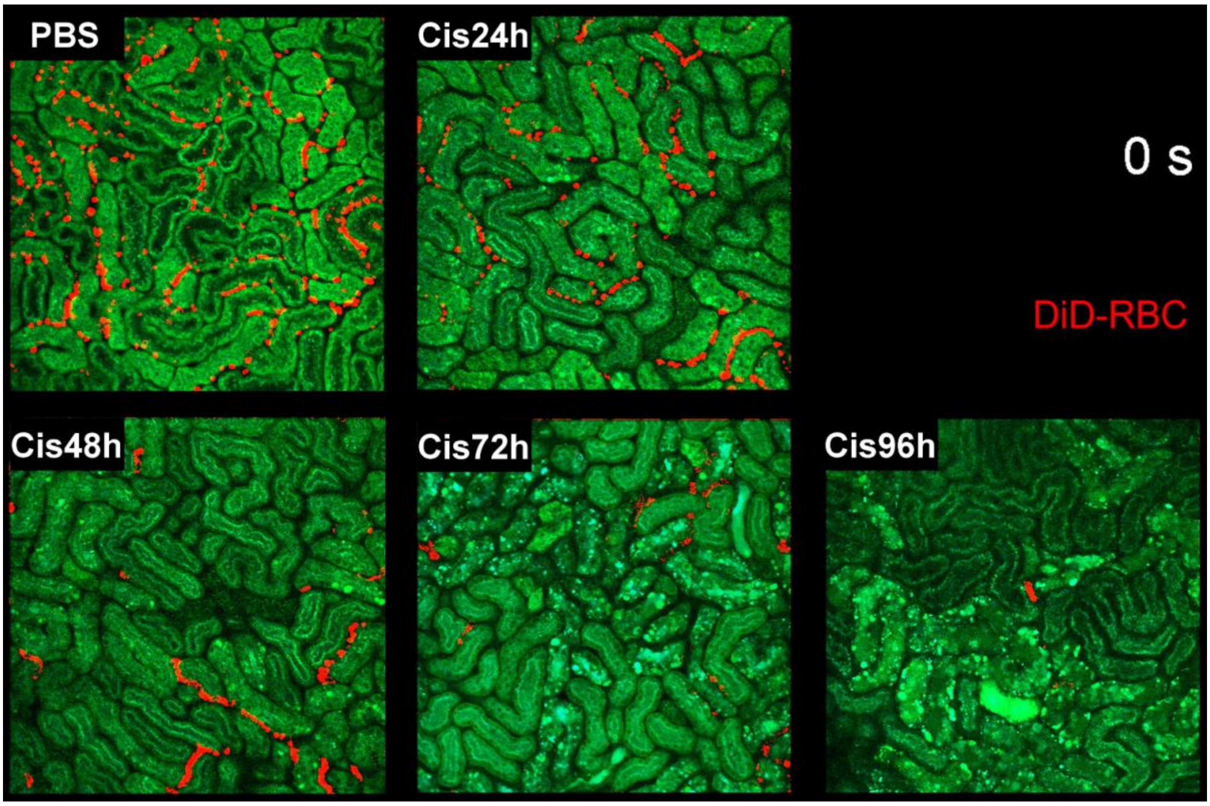

Supplemental Video 5. Mapping of the functional capillary ratio using cellular angiography in peritubular microcirculation in cisplatin-induced AKI

DiD-labeled erythrocytes (red) were intravenously injected into C57BL/6N mice via a tail vein catheter. Mapping of functional capillaries using cellular angiography was performed. This video corresponds to Figure 3A. Time is marked as SS (seconds).

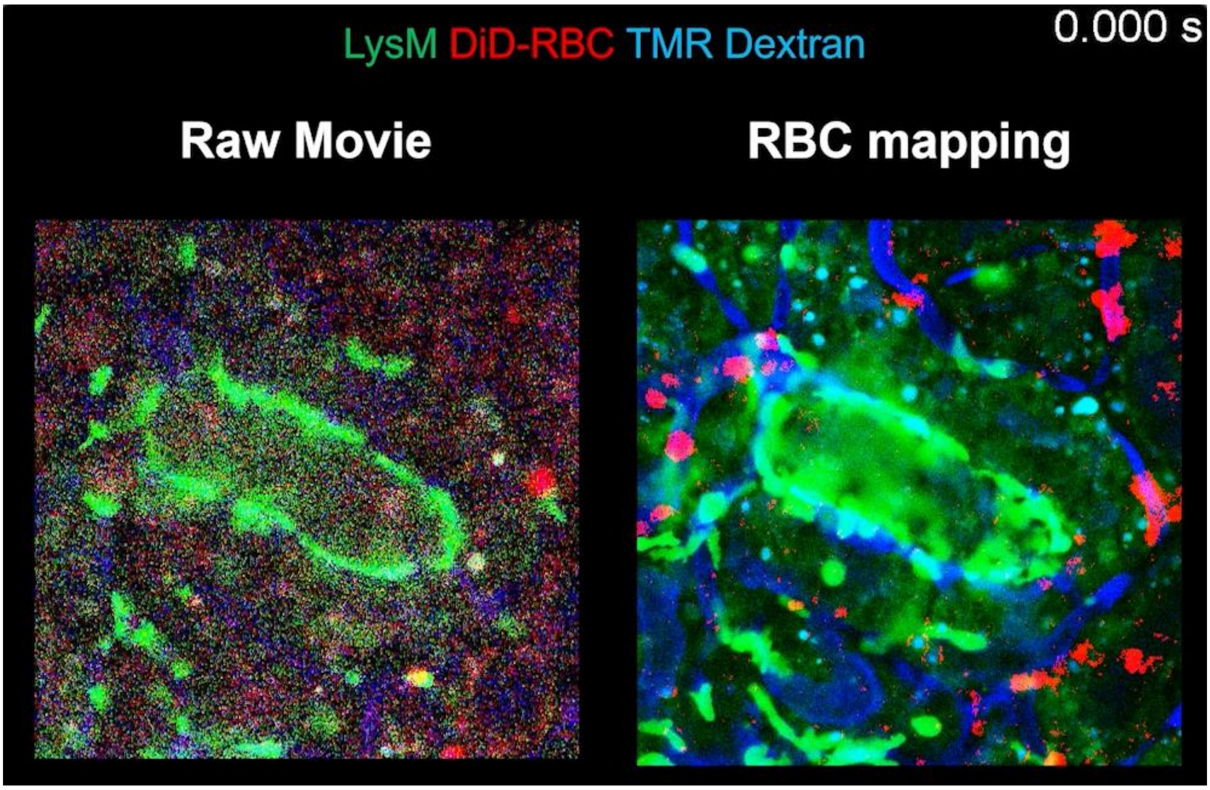

Supplemental Video 6. Neutrophil infiltration does not impede surrounding microcirculation in cisplatin-induced AKI

TMR–dextran (blue) and DiD-labeled erythrocytes (red) were intravenously injected into LysM$^{GFP/+}$ (green) mice via a tail vein catheter. From the raw Video (left), mapping of erythrocytes (right) was generated. Neutrophil infiltration in tubules does not interfere with the nearby microcirculation. This video corresponds to Figure 4D. Time is marked as SS.SSS (seconds).

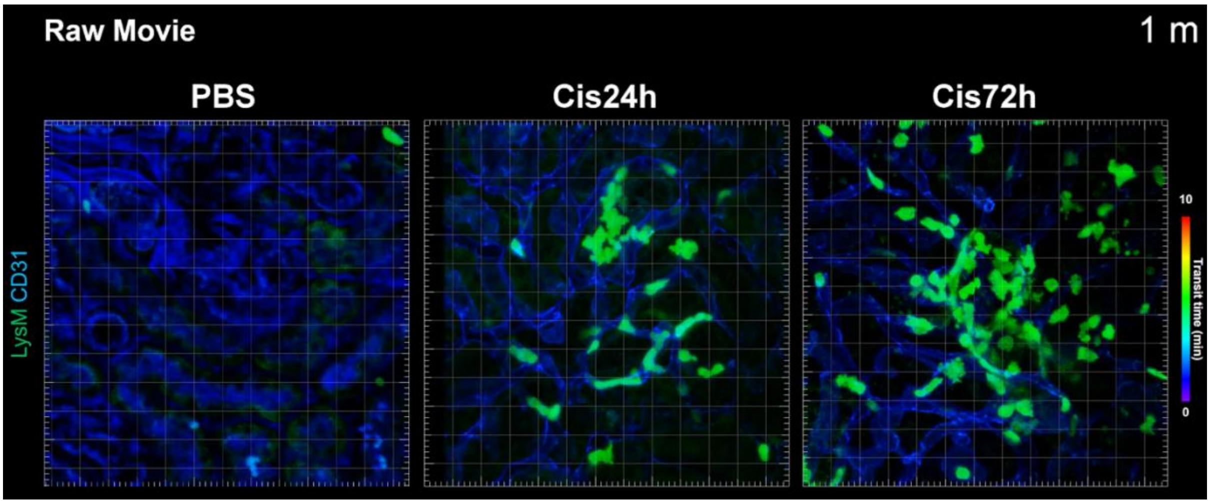

Supplemental Video 7. Neutrophil dynamics in proximal tubules in cisplatin-induced AKI

10 minutes of 1 minute time-interval intravital kidney imaging of C57BL/6N mouse was performed to assess neutrophil behaviors in PBS, Cis 24h, and Cis 72h group. The video consisted with the raw movie, LysM track, and merged movie. Dynamics of LysM+ (green) cells near tubule and peritubular vessel (CD31, blue) was identified and the trajectory of individual neutrophil was visualized. Color bar represents the transit time of the track of LysM+ (0-10 minutes). This video corresponds to Figure 4F. Time is marked as MM (minutes).

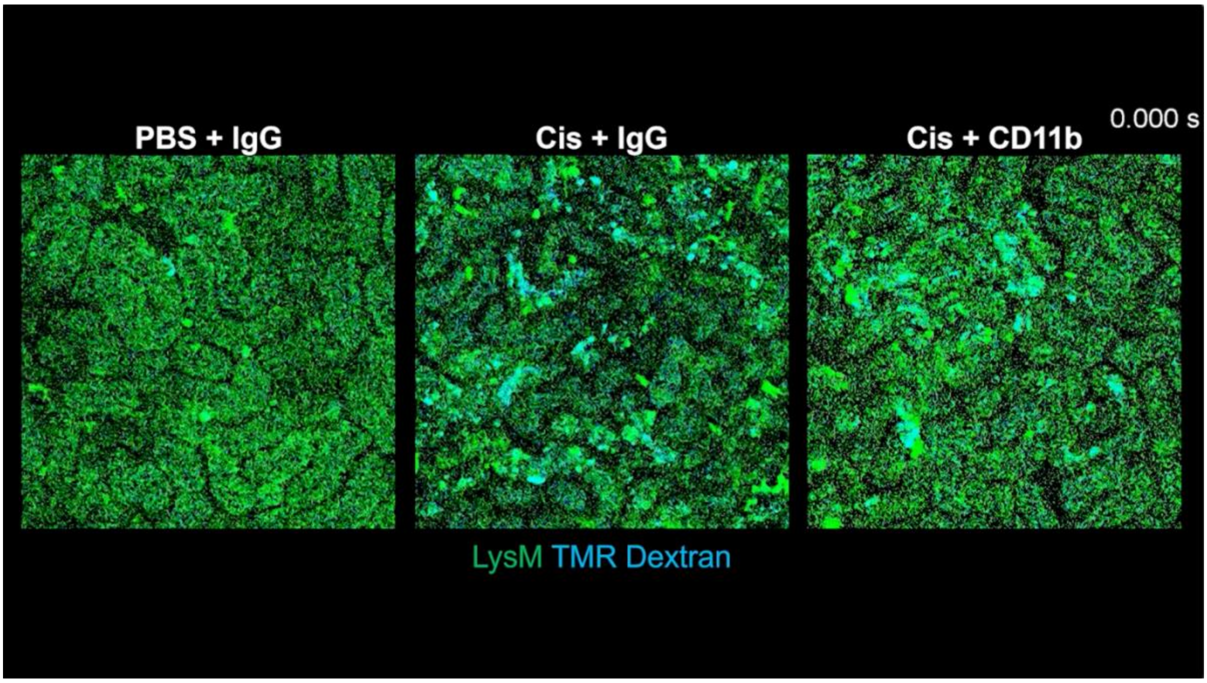

Supplemental Video 8. Fluorescence angiography of peritubular microcirculation after CD11b antibody treatment for cisplatin-induced AKI

TMR–dextran (blue) was intravenously injected into LysM$^{GFP/+}$ mice via a tail vein catheter. Peritubular microcirculation, as quantified by time to peak and perfusion area, was improved in the CD11b antibody treatment group (Cis+CD11b). This Video corresponds to Figure 5A. Time is marked as SS.SSS (seconds).

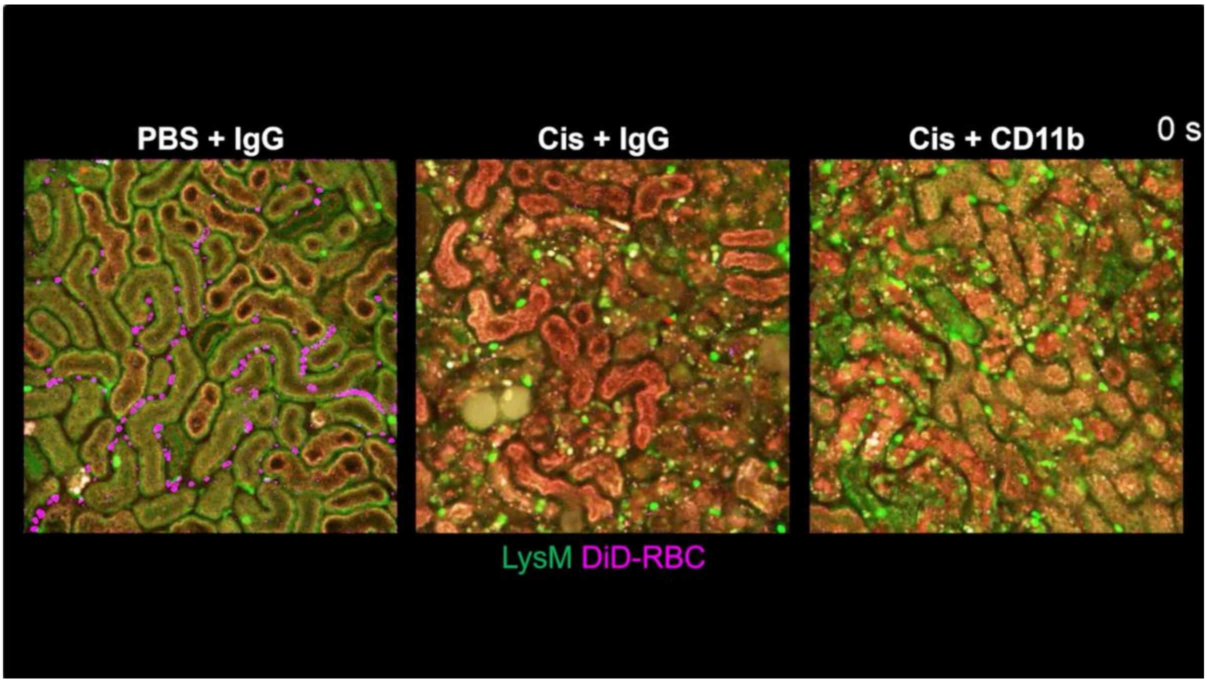

Supplemental Video 9. Cellular angiography of peritubular microcirculation after CD11b antibody treatment for cisplatin-induced AKI

DiD-labeled erythrocytes (magenta) were intravenously injected into LysM$^{GFP/+}$ mice via a tail vein catheter. Mapping of functional capillaries using cellular angiography indicated improvement in the CD11b antibody treatment group (Cis+CD11b). This video corresponds to Figure 5E. Time is marked as SS (seconds).
